# Retraction: Late Complications of Clinical *Clostridium Histolyticum* Collagenase Use in Dupuytren's Disease

**DOI:** 10.1371/annotation/81e8574c-71c3-4a76-b1aa-e3c7b058af69

**Published:** 2012-11-05

**Authors:** Warren M. Rozen, Yasith Edirisinghe, John Crock

After the publication of the article, a number of concerns were identified in relation to the results and information reported in the article. The concerns are as follows:

1) One of the two patients did not receive the intervention involving an injection of Clostridium histolyticum collagenase as reported in the article.

2) The statement regarding ethical approval in the article is inaccurate. The study did not receive ethical approval. On the basis of the information reported in the article, the editors consider that the study involved the prospective collection of outcome data and as a result, approval by an ethics committee would have been required.

In the light of the concerns outlined, the authors and the editors retract this publication.

